# Antimicrobial activity of the ethanolic extract of *Guettarda sericea*against oral streptococci

**DOI:** 10.1186/1753-6561-8-S4-P54

**Published:** 2014-10-01

**Authors:** Edson Teixeira, Érica Rabelo, Bruno Silva, Luiz Nascimento Neto, Francisco Arruda, Hélcio Santos, Maria Albuquerque, Paulo Bandeira, Kyria Nascimento, Benildo Cavada, Jorge Martins

**Affiliations:** 1 Federal University of Ceará, Brazil; 2 Acaraú Valley State University, Sobral, Ceará, Brazil; 3 Federal University of Pelotas, Brazil

## Background

The genus *Guettarda *(Rubiaceae) comprises plants widely distributed in tropical areas [1]. Regarding the *Guettarda sericea *species, the literature shows that there is a lack of botanical and phytochemicals studies [2]. Thus, the present study aimed to evaluate the antibacterial effect of ethanol extract of leaves of *G. sericea *(EEFGS) on the growth of *Streptococcus oralis *ATCC 10557 and *S. salivarius *ATCC 7073 in both the planktonic and biofilms states.

## Methods

Different methods were employed to verify the antimicrobial potential. Among these are the determination of minimum inhibitory concentration (MIC) determination of the death curve and evaluation of minimum bactericidal concentration (MBC) [3]. Furthermore, quantification of biomass and the number of viable cells of the biofilm were performed [4]. The negative and positive controls used in all assays were respectively 4% DMSO and chlorhexidine gluconate. To determine the toxicity of EEFGS, it was used the toxicity test on *Artemia *nauplii [5].

## Results and conclusions

The data showed that the extract has a remarkable antimicrobial effect, able to inhibit the growth of planktonic and development of biofilms of *S. oralis *strain until the concentration of 62.5 μg.mL^-1^. Regarding the toxicity, it was observed that death of *Artemia *nauplii occurred at higher concentrations than those that exhibited antibacterial effect. It can be concluded that EEFGS can be used as agents for the control of biofilm formation of *S. oralis*. In addition, complementary methodologies that seek purification of the active compounds and their cytotoxic effects on eukaryotic cells need to be held, aiming its use as an herbal agent.
